# Effects of glucagon‐like peptide 1 receptor agonists on testicular dysfunction: A systematic review and meta‐analysis

**DOI:** 10.1111/andr.70022

**Published:** 2025-03-19

**Authors:** Gianmaria Salvio, Alessandro Ciarloni, Nicola Ambo, Monia Bordoni, Michele Perrone, Silvia Rossi, Giancarlo Balercia

**Affiliations:** ^1^ Endocrinology Clinic, Department of Clinical and Molecular Sciences Polytechnic University of Marche Ancona Italy

**Keywords:** GIP, GLP‐1, GLP‐1RA, hypogonadism, obesity, overweight, testosterone

## Abstract

**Background:**

Hypogonadism and infertility are two conditions that are heavily affected by overweight and obesity in the male patient. Glucagon‐like peptide 1 agonists (GLP‐1RAs) are a recently introduced class of antidiabetic drugs with powerful weight‐loss effect; this may induce an indirect positive effect on the testicular function. Nevertheless, recent evidence also suggests a potential direct influence of these molecules on the gonadal function.

**Objectives:**

Our study aims at evaluating the effects of GLP‐1RAs on hormone secretion in male patients and comparing their impact on the testicular function with other antidiabetic agents or weight‐lowering drugs.

**Materials and methods:**

A literature search was conducted using PubMed, EMBASE, and Scopus database to assess the effects of GLP‐1RAs on hormone levels, sperm parameters, and erectile function in overweight and obese men. Before–after analysis and comparison between therapy with GLP‐1RAs and other treatment regimens were performed.

**Results:**

Seven studies (*n* = 680) were included in the quantitative analysis. Treatment with GLP‐1RAs produced a significant increase in total serum testosterone (TT), with a standardized mean difference of 1.39 ng/mL (95% confidence interval: 0.70, 2.09; *p* < 0.0001). Free serum testosterone (FT), sex hormone‐binding globulin (SHBG), luteinizing hormone (LH), and follicle‐stimulating hormone (FSH) showed similar increase, while weight, body mass index (BMI), waist circumference (WC), and glycated hemoglobin (HbA1c) decreased. Meta‐regression showed a significant negative correlation between standardized mean difference in TT levels before–after treatment and percentage change in weight and BMI. When compared with other treatment options, GLP‐1RAs showed a comparable effect on serum androgens, but greater BMI reduction and increase in serum gonadotropins and indexes of the erectile function.

**Conclusion:**

Our systematic review and meta‐analysis suggest a possible role for GLP‐1RAs in the therapy of functional hypogonadism related to overweight and obesity, while also promoting weight loss. The limitations of the current literature do not allow to demonstrate a direct action of GLP‐1RAs on the testicular function.

## 1 INTRODUCTION

Male hypogonadism and infertility are two common conditions in which overweight and excess adipose tissue may have an etiopathogenetic role.^1^, ^2^, ^3^ Indeed, aromatase activity of adipose tissue—especially when the latter is present in excess—leads to higher oestradiol levels and subsequent suppression of gonadotropic secretion. The latter results in low testosterone levels and impaired semen quality (functional hypogonadism).^3^, ^4^Overweight is associated with chronic inflammation and high scrotal temperature, which can directly impair the sperm cells function.^1^, ^2^, ^5^ Furthermore, obesity is often associated with unhealthy lifestyle habits as smoking, alcohol abuse, and sedentary lifestyle, which can also contribute to the impairment of male fertility.^1^, ^2^

Glucagon like peptide‐1 (GLP‐1) receptor agonists (GLP‐1RAs) are the most recently introduced class of antidiabetic drugs, which display greater effects on blood glucose control compared with other oral antidiabetic agents and a very low hypoglycemic risk.^6^ They also produce positive effects on dyslipidaemia and hypertension,^7^, ^8^ leading to significant cardiovascular risk reduction.^7^, ^8^ Interestingly, GLP‐1RAs also act as effective weight‐lowering agents, modulating appetite and energy intake through delayed gastric emptying and direct effect on the brain centers of satiety.^9^, ^10^, ^11^ Indeed, following peripheral administration in murine models, liraglutide binds to GLP‐1 receptor (GLP1‐R) on arcuate nucleus neurons expressing proopiomelanocortin (POMC) and cocaine‐ and amphetamine‐regulated transcript (CART) in the hypothalamus, thus increasing their activity, and inhibiting the activity of neurons expressing neuropeptide Y (NPY) and agouti‐related peptide (AgRP), relevant for appetite regulation.^12^ As their efficacy on weight loss is established, these drugs are currently used in the pharmacological treatment of obesity,^13^ and may contribute to improve testicular function in subjects suffering from functional hypogonadism.

Furthermore, pre‐clinical evidence suggests a direct effect of GLP‐1 on the reproductive function. In another study on murine models, GLP‐1R knockout animals showed conserved fertility, with delayed puberty and lower adrenal gland volumes in females, and volumetric reduction of adrenal glands, testicles and seminal vesicles in males.^14^ The discovery of GLP‐1R on Leydig and Sertoli cells and on spermatozoa of animals and humans further supports this hypothesis.^15^, ^16^ Interestingly, GLP‐1 seems to also have a role in stimulating metabolism of testicular cells by increasing lactate levels without stimulating glucose intake.^16^ Moreover, the interaction between GLP‐1 and GLP‐1R seems to improve sperm motility by a direct non‐insulin mediated mechanism in vitro.^16^ Finally, other in vitro studies suggest an anti‐inflammatory effect of GLP‐1 that may counteract the well‐known negative effects of oxidative stress related to chronic inflammation on male infertility.^16^, ^17^

A modulation of the testicular function seems to be possible in humans as well. Indeed, although continuous intravenous infusion of GLP‐1 in healthy young males did not affect total testosterone levels, an alteration of its pulsatile secretion pattern was observed.^18^ Despite this, the effect of these drugs on the hormone balance of individuals with functional hypogonadism is still unclear.

The aim of the present meta‐analysis is to investigate the potential role of GLP‐1RAs in the treatment of male hypogonadism and infertility.

## 2 MATERIALS AND METHODS

This study was conducted following the guidelines of The Preferred Reporting Items for Systematic reviews and Meta‐Analyses (PRISMA) statement.^19^ The research was registered on PROSPERO (https://www.crd.york.ac.uk/prospero/) with number CRD42024497949.

### 2.1 Search strategy

A systematic search was conducted through Scopus, PubMed, and EMBASE databases from June to August 2024. The terms “GLP‐1,” “liraglutide,” “exenatide,” “dulaglutide,” “semaglutide,” “lixisenatide,” and “glucagon‐like peptide 1” were combined with “hypogonadism,” “testosterone,” “testicular,” “sperm,” “erectile dysfunction,” and “infertility” (see Supporting Information for full research strings). The search was carried out independently by two authors (GS and AC), and disagreements were resolved through discussion with a third author (GB).

### 2.2 Selection criteria

The eligible studies were selected following the PICO model: population (P: overweight/obese men), intervention (I: GLP‐1RA administration), comparison (C: same patients before–after treatment/untreated subjects), outcome (O: hormone levels, sperm parameters, erectile function). Subjects treated with drugs other than GLP‐1RAs were considered as untreated. Randomized controlled trials, retrospective and prospective cohort studies were included. No language restrictions were applied.

### 2.3 Data extraction and quality assessment

Two authors (GS and AC) performed data extraction, which was subsequently verified by a third author (GB). The following data were collected: first author, year, country, study design, characteristics of patients, type and dosage of GLP‐1RA, associated treatments, type of controls, length of follow‐up, age, weight, body mass index (BMI), waist circumference (WC), serum levels of glycated hemoglobin (HbA1c), total testosterone (TT), free testosterone (FT), sex hormone binding globulin (SHBG), estradiol, luteinizing hormone (LH), follicle‐stimulating hormone (FSH), and International Index of Erectile Function (IIEF) score. The quality of evidence (QoE) was assessed using the New‐Ottawa scale (NOS) for cohort studies.^20^ Two researchers (GS and AC) performed the QoE and a third author (GB) verified their entries.

### 2.4 Statistical analysis

The analysis was performed using RevMan software v. 5.4.1 (Cochrane Collaboration, Oxford, UK) and Comprehensive Meta‐Analysis v. 3.7 (Biostat Inc., Englewood, USA). Standardized mean difference (sMD) and 95% confidence intervals (CIs) were calculated to compare outcome measures after treatment. *I* ^2^ statistic was applied to inspect heterogeneity, with *I* ^2^ > 50% and *p* < 0.1 indicating high between‐study heterogeneity. If significant heterogeneity emerged, meta‐analysis was performed using a random‐effects model. Otherwise, a fixed‐effects model was used. If more than one longitudinal measurement was available, the longest follow‐up from each study was selected (as recommended in the Cochrane Handbook for Systematic Reviews for Interventions^21^). Publication bias was assessed by funnel plot asymmetry as well as Egger's test. To investigate the source of heterogeneity, sensitivity analysis (omitting each single study to explore its effect on the overall meta‐analysis) was carried out, and subgroup analysis was conducted by differentiating subjects treated with other antidiabetic drugs and those treated with hormones. In addition, univariate meta‐regression was also performed to explore the effects of percentage change in weight, BMI, WC, and HbA1c levels on male hormone levels. Statistical significance was set at 0.05.

## 3 RESULTS

### 3.1 Study selection

**FIGURE 1:**
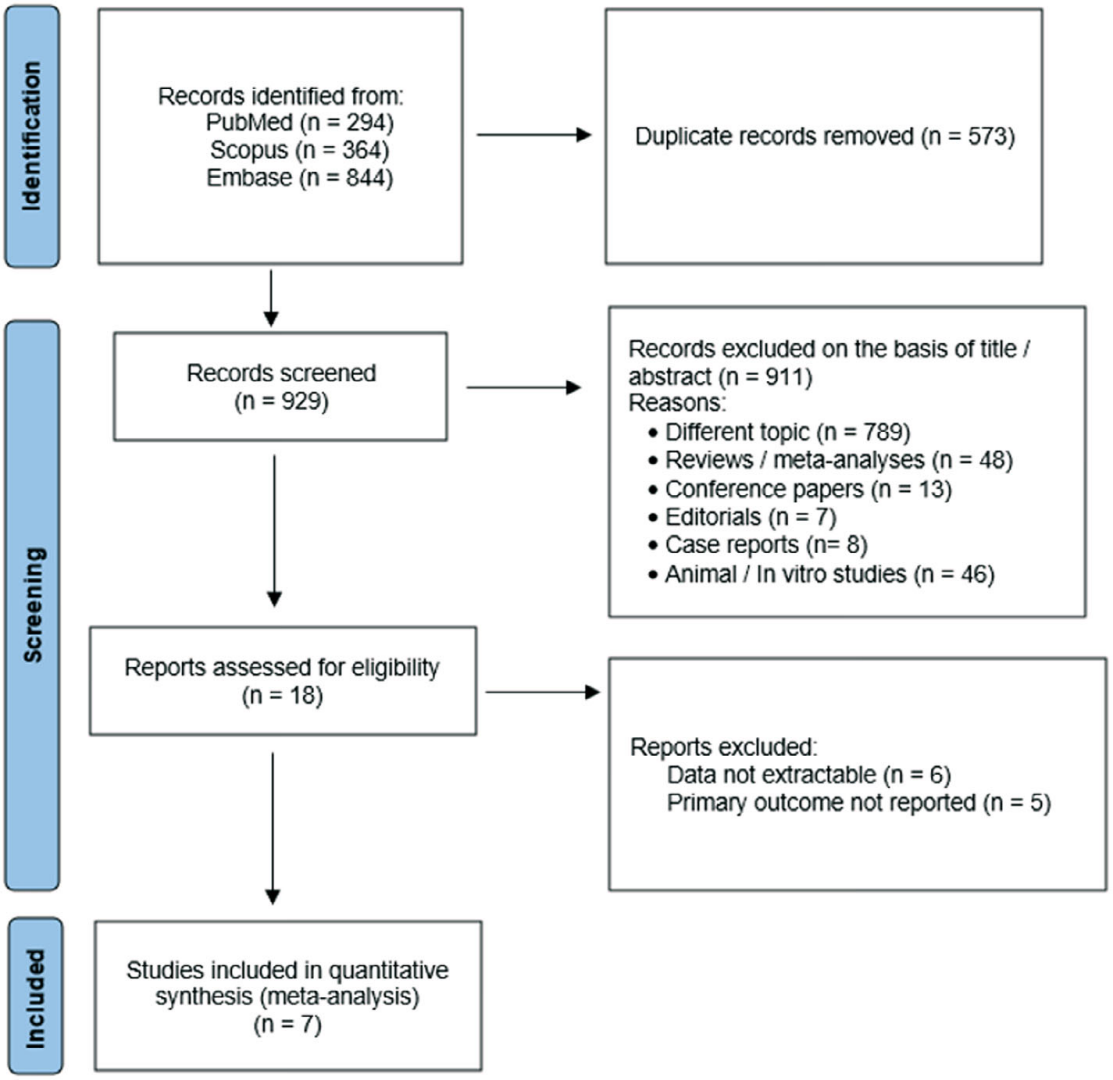
Preferred reporting items for systematic review and meta‐analysis protocols (PRISMA‐P) flowchart.

Using the above‐mentioned search strategy, 1502 abstracts were found. After the removal of 573 duplicates, 929 articles were screened. Out of these, 911 were identified by title or abstracts as papers on other topics, review articles, editorials, case reports, animal or in vitro studies, guidelines, or congress papers. Of the remaining 18 full‐text articles assessed for eligibility, seven^22^, ^23^, ^24^, ^25^, ^26^, ^27^, ^28^ were included in the present meta‐analysis (Figure 1). The 11 studies not included in the quantitative analysis, with reasons for exclusion, are shown in Table S1. Since only two studies evaluated the effect of GLP‐1RAs on semen parameters,^28^, ^29^ this assessment was not performed and only the effect of GLP‐1RAs on hormone levels and erectile function are shown. Of the included studies, three were retrospective cohort,^23^, ^24^, ^26^ two were prospective cohort,^22^, ^24^, ^25^ and one was a prospective randomized open‐label study.^27^ All the studies included in our meta‐analysis were controlled except for one^25^ that was a single‐arm cohort study. The main characteristics of the included studies are shown in Table 1.

**TABLE 1:** Characteristics of the included studies.

First author	Year	Country	Study design	Sample size (no. treated)	Prevalence of diabetes	Prevalence of overweight or obesity	Prevalence of hypogonadism	Age (years) Mean ± SD median (IQR)	BMI (kg/m^2^)	Drug and dose	Additional drugs	Controls	Follow‐up time (months)
Shao^22^	2018	China	Prospective cohort, controlled vs. other antidiabetics	176 (90)	100%	100%	NA	GLP‐1RAs 43 ± 8,5 Ctrl 44 ± 7	GLP‐1RAs 30.52 ± 3.34 Ctrl 30.29 ± 2.91	Exenatide SC 10 mcg BID	Metformin 1 g/die	Glimepiride 4 mg/die + Metformin 1000 mg/die	3
Giagulli^23^	2015	Italy	Retrospective cohort, controlled vs. no additional treatment	Group 1 30 (16) Group 2 13 (10)	100%	100%	100%	Group 1 53.5 ± 4.4 Group 2 50.6 ± 4.3	Group 1 34.2 ± 2.4 Group 2 34.7 ± 2.3	Liraglutide SC 1.2 mcg/die	Testosterone Undecanoate 1000 mg/12 weeks + Metformin 2–3 g/die	Testosterone Undecanoate 1000 mg/12 weeks + Metformin 2–3 g/die	12
Lisco^24^	2023	Italy	Retrospective cohort, controlled vs. no additional treatment	108 (63)	100%	100%	NA	GLP‐1RAs 59 (56–65) Ctrl 60 (58–64)	GLP‐1RAs 34 ± 1.7 Ctrl 33.7 ± 1.7	Liraglutide SC 1.2 mg/die or Dulaglutide SC 1,5 mg/weekly	Metformin 500 mg up to maximum tolerated dosage (mean dosage 2000 mg/die)	Metformin 500 mg up to maximum tolerated dosage (mean dosage 2000 mg/die)	12
Graybill^25^	2020	USA	Prospective cohort, single‐arm treatment	51 (51)	100%	NA	NA	57.6 ± 8.8	34.9 ± 5.3	Exenatide ER SC 2 mg/weekly	Any antiadiabetic drug	NA	12
Giagulli^26^	2020	Italy	Retrospective cohort, controlled vs. no additional treatment	71 (30)	100%	100%	NA	50.3 ± 3.3	33.7 ± 1.7	Dulaglutide SC 1,5 mg/weekly (14 patients) and Liraglutide SC 1.2 mg/die (16 patients)	Metformin 2–3 g/die	Metformin 2–3 g/die	12
Jensterle^27^	2019	Slovenia	Randomized open‐label, controlled vs. hormone treatment	30 (15)	36.7%	100%	100%	GLP‐1RAs 43.9 ± 11.6 Ctrl 49.3 ± 9.8	GLP‐1RAs 43.2 ± 7.5 Ctrl 39 ± 9	Liraglutide SC 3.0 mg/die	–	Transdermal testosterone gel 1% 50 mg/die	4
La Vignera^28^	2023	Italy	Prospective cohort, controlled vs. hormone treatment	110 (35)	0%	100%	100%	26 ± 6	Group A 35 ± 3 Group B 36 ± 3 Group C 33 ± 2	Liraglutide SC 3 mg/week (Group A)		Urofollitropin 150 UI/3 times a week + chg. 2000 UI twice a week (Group B) or transdermal testosterone gel 2% 60 mg/day (Group C)	4

Abbreviations: IQR, iterquartile range; NA, not available.

### 3.2 Risk of bias assessment

Risk of bias was assessed for 6 out of 7 studies (Table 2), because in one case^25^ no control group was available. Three studies^22^, ^24^, ^27^ presented good quality, while the other three showed fair^23^ and poor quality,^26^, ^28^ respectively.

**TABLE 2:** Risk of bias assessment.

	Selection					Outcome				
Studies	Representativeness of the present cohort	Selection of non‐exposed	Ascertaiment of exposure	Outcome not present at start	Comparability	Assessment of outcome	Adequate follow‐up length	Adequate follow‐up	Score	Quality
Shao et al. 2018^22^	★	★	★	★	★★	★	★	★	9	Good
Giagulli et al. 2015^23^	–	★	–	★	★	★	★	–	5	Fair
Lisco et al. 2023^24^	★	★	–	★	★★	★	★	–	7	Good
Giagulli et al. 2020^26^	–	★	★	★	–	★	★	–	5	Poor
Jensterle et al. 2019^27^	★	★	–	★	★★	★	★	★	8	Good
La Vignera et al. 2023^28^	–	★	–	★	–	★	★	–	4	Poor

### 3.3 Before–after analysis

**FIGURE 2:**
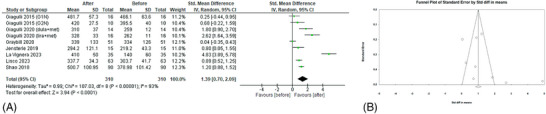
(A) Forest plot of serum TT levels of patients before and after GLP‐1RAs administration; (B) funnel plot of the included studies. GLP‐1RAs, glucagon‐like peptide 1 receptor agonists; TT, total testosterone.

At first, a before–after analysis to evaluate the effects of GLP‐1RAs on serum hormone levels was executed. All the seven studies^22^, ^23^, ^24^, ^25^, ^26^, ^27^, ^28^ evaluated the effects of GLP‐1RAs on TT levels (Figure 2A), showing a significant increase after the administration of these drugs (sMD 1.39; 95% CI: 0.70, 2.09; *p* < 0.0001). Heterogeneity was high (*I* ^2 ^= 93%) and no publication bias were found (Egger's test, *p* = 0.21; Figure 2B). Sensitivity analysis identified the study of La Vignera et al.^28^ as the major source of heterogeneity, but after its removal no significant changes emerged (sMD 0.96; 95% CI: 0.48, 1.44; *p* < 0.0001, *I* ^2 ^= 83%).

**FIGURE 3:**
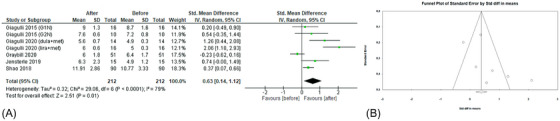
(A) Forest plot of serum FT levels of patients before and after GLP‐1RAs administration; (B) funnel plot of the included studies. FT, free testosterone; GLP‐1RAs, glucagon‐like peptide 1 receptor agonists.

The effects of GLP‐1RAs administration on FT levels were evaluated in five studies^22^, ^23^, ^25^, ^26^, ^27^ (Figure 3A). A significant increase in FT levels emerged (sMD 0.63; 95% CI: 0.14, 1.12; *p* = 0.01), with high heterogeneity (*I* ^2 ^= 79%). Egger's test (*p* = 0.14) and Funnel plot (Figure 3B) showed no publication bias. Concerning sensitivity analysis, after the removal of the study by Giagulli et al.,^26^ heterogeneity decreased but the effects of GLP‐1RAs on FT levels were no longer significant (sMD 0.25; 95% CI: −0.09, 0.60; *p* = 0.15; *I* ^2^ = 52%).

**FIGURE 4:**
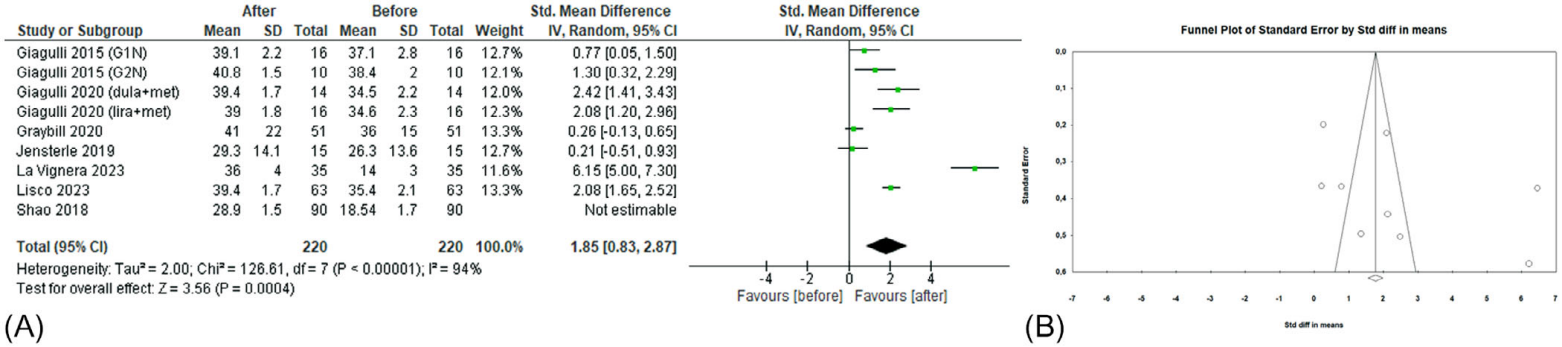
(A) Forest plot of serum SHBG levels of patients before and after GLP‐1RAs administration; (B) funnel plot of the included studies. GLP‐1RAs, glucagon‐like peptide 1 receptor agonists; SHBG, sex hormone‐binding globulin.

All the included studies^22^, ^23^, ^24^, ^25^, ^26^, ^27^, ^28^ reported data on SHBG levels before–after treatment with GLP‐1RAs (Figure 4A). A slight but significant increase in SHBG levels was shown (sMD 2.39; 95% CI: 1.01, 3.77; *p* = 0.0007), with high heterogeneity (*I* ^2 ^= 97%). Egger's test (*p* = 0.20) and Funnel plot (Figure 4B) did not show publication bias. A slight decrease in heterogeneity emerged after the removal of the study by Shao et al.,^22^ but results were unchanged (sMD 1.85; 95% CI: 0.83, 2.87; *p* = 0.0004; *I* ^2^ 94%).

**FIGURE 5:**
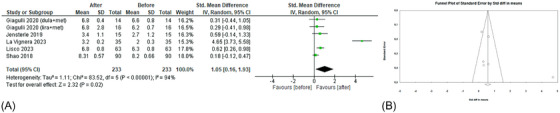
(A) Forest plot of serum LH levels of patients before and after (GLP‐1RAs) administration; (B) funnel plot of the included studies. GLP‐1RAs, glucagon‐like peptide 1 receptor agonists; LH, luteinizing hormone.

Concerning serum gonadotropin levels, five studies^22^, ^24^, ^26^, ^27^, ^28^ showed that the administration of GLP‐1RAs led to an increase in serum LH levels (sMD 1.05; 95% CI: 0.16, 1.93; *p* = 0.02) (Figure 5A). No publication bias was shown by Funnel plot (Figure 5B) and Egger's test (*p* = 0.25). Excluding the study by La Vignera et al.,^28^ heterogeneity decreased dramatically (*I* ^2 ^= 0%), but the effects remained significant (sMD 0.36; 95% CI: 0.16, 0.56; *p* = 0.0003).

**FIGURE 6:**
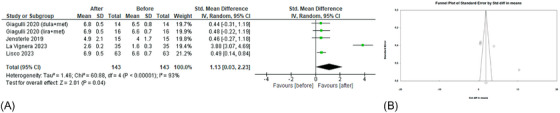
(A) Forest plot of serum FSH levels of patients before and after GLP‐1RAs administration; (B) funnel plot of the included studies. FSH, follicle‐stimulating; GLP‐1RAs, glucagon‐like peptide 1 receptor agonists.

FSH levels before and after the administration of GLP‐1RAs were investigated in four studies.^24^, ^26^, ^27^, ^28^ As for LH levels, FSH levels significantly increased (sMD 1.13; 95% CI: 0.03, 2.23; *p* = 0.04) (Figure 6A). Notably, high heterogeneity (*I* ^2^ = 93%) was present and possible publication bias was suggested by Funnel plot (Figure 6B) and Egger's test (*p* = 0.017). Notably, the exclusion of the study by La Vignera et al.^28^ led to a marked decrease in heterogeneity, leaving the effects on FSH levels unchanged (sMD 0.48; 95% CI: 0.21, 0.75; *p* = 0.0005; *I* ^2^ 0%).

**FIGURE 7:**
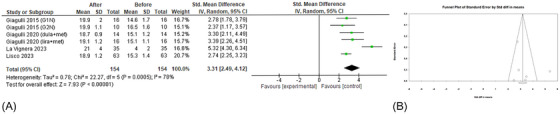
(A) Forest plot of IIEF scores of patients before and after GLP‐1RAs administration; (B) funnel plot of the included studies. GLP‐1RAs, glucagon‐like peptide 1 receptor agonists; IIEF, International Index of Erectile Function.

As an indirect index of the gonadal function, the presence of erectile dysfunction (ED) was investigated. The analysis of four studies^23^, ^24^, ^26^, ^28^ showed that GLP‐1RAs administration led to a significant increase in IIEF score (sMD 3.31; 95% CI: 2.49, 4.12; *p* < 0.00001; *I* ^2^ = 78%) (Figure 7A). No publication bias emerged (Egger's test, *p* = 0.42; Figure 7B). After the removal of the study by La Vignera et al.,^28^ heterogeneity decreased, but the effects of GLP‐1RAs on the erectile function were unchanged (sMD 2.84; 95% CI: 2.47, 3.21; *p* < 0.00001; *I* ^2^ = 0%).

As secondary outcomes, the effects of GLP‐1RAs administration on weight, BMI, WC, and HbA1c levels were evaluated. All of the above‐mentioned parameters showed a significant improvement. In detail, a mean decrease of 8.54 kg in body weight (*p* < 0.00001), 2.16 kg/m^2^ in BMI (*p* = 0.0002), and 7.78 cm in WC (*p* < 0.00001) confirmed the positive effects of GLP‐1RAs as weight‐lowering agents, whereas their antidiabetic effects were underlined by the mean decrease of 1.15% in HbA1c levels (*p* < 0.00001) (Figure S1).

### 3.4 Meta‐regression analysis

**FIGURE 8:**
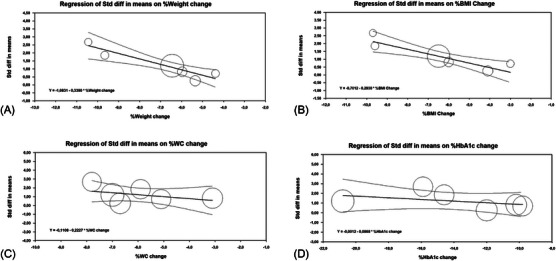
Scatter plot showing the effect of percentage weight change (A), BMI change (B), WC change (C), and HbA1c change (D) on the standardized mean of difference of total testosterone levels before‐after GLP‐1RAs treatment. BMI, body mass index; GLPA‐1a, glucagon‐like peptide‐1 agonists; HbA1c, glycated hemoglobin; WC, waist circumference.

The meta‐regression analysis showed that sMD in TT levels before–after GLP‐1RAs treatment increased as a function of weight and BMI loss percentage (Figure 8A,B and Table 3). On the other hand, mean difference in TT levels were independent of the percentage change in WC and HbA1c (Figure 8C,D).

**TABLE 3:** Meta‐regression analysis.

Mean difference	Moderator	*R*‐squared	Coefficient (95% CI)	Standard error	*p*‐value
TT	%Weight change	1.00	−0.339 (−0.501, −0.177)	0.082	<0.0001
	%BMI change	1.00	−0.293 (−0.433, −0.152)	0.072	<0.0001
	%WC change	0.00	−0.222 (−0.626, 0.181)	0.206	0.279
	%HbA1c change	0.00	−0.0868 (−0.260, 0.086)	0.088	0.326
FT	%Weight change	0.91	−0.273 (−0.435, −0.111)	0.083	0.001
	%BMI change	0.57	−0.212 (−0.387, −0.037)	0.089	0.017
	%WC change	0.00	−0.103 (−0.493, 0.287)	0.199	0.606
	%HbA1c change	0.00	−0.012 (−0.172, 0.148)	0.082	0.882
SHBG	%Weight change	0.00	−0.159 (−1.128, 0.810)	0.495	0.748
	%BMI change	0.00	−0.211 (−1.051, 0.629)	0.429	0.623
	%WC change	0.07	−0.694 (−1.951, 0.563)	0.641	0.279
	%HbA1c change	0.90	−0.507 (−0.693, −0.322)	0.095	<0.0001

Abbreviations: BMI, body mass index; FT, free testosterone; HbA1c, glycosylated hemoglobin; SHBG, sex hormone‐binding globulin; TT, total testosterone; WC, waist circumference.

Similarly, sMD in FT levels before–after treatment were dependent on percentage change in weight and BMI but not of WC and HbA1c (Table 3).

Interestingly, sMD in SHBG levels before–after treatment revealed to be independent of percentage change in weight, BMI, and WC, whereas a significant relationship with percentage change in HbA1c emerged (Table 3).

### 3.5 Comparison analysis

Comparison analysis was conducted by comparing subjects treated with GLP‐1RAs and control groups on clinical parameters and hormone levels obtained after the treatment. Of the included studies, all except one^25^ presented a control group and were included in comparison analysis. Subgroup analysis was conducted to compare treatment with antidiabetics/no treatment versus hormone replacement therapy. In the study by Giagulli et al.,^23^ metformin and testosterone undecanoate were administered in both the GLP‐1RAs and control group, so the latter was considered as “no treatment.” When compared with controls, patients who underwent treatment with GLP‐1RAs showed a more pronounced decrease in BMI (MD −2.08; 95% CI: −2.54, −1.63; *p* < 0.00001; *I* ^2^ = 30%),^22^, ^24^, ^26^, ^27^ and difference between groups was not significant (*p* = 0.59). The effects on weight,^22^, ^23^, ^24^, ^26^, ^27^ WC,^22^, ^26^, ^27^ and HbA1c levels^22^, ^24^, ^26^, ^27^ were similar (Figure S2).

**FIGURE 9:**
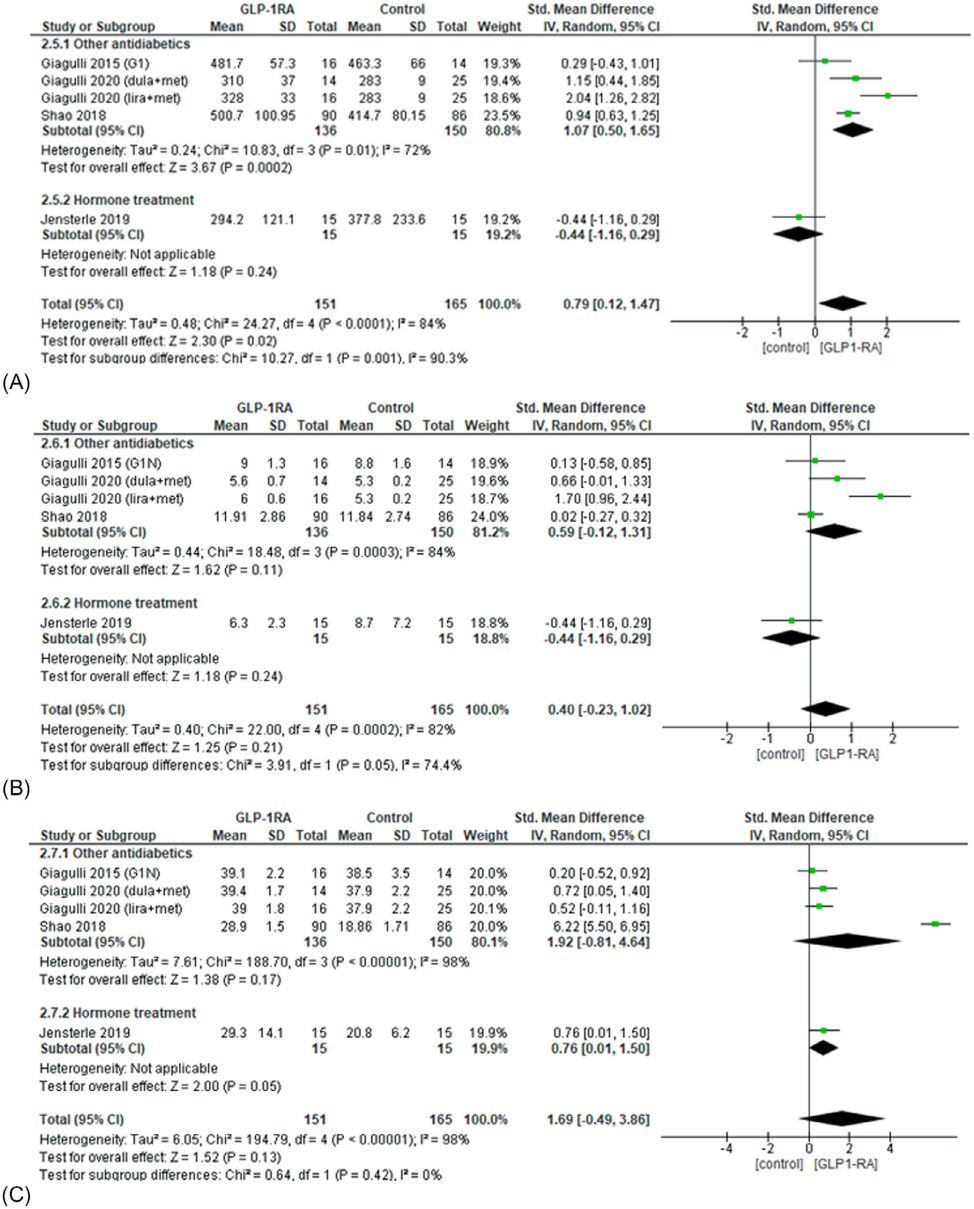
Subgroup analysis and forest plot of TT (A), FT (B), and SHBG (C) levels in patients treated with GLP‐1RAs compared with controls. GLP‐1RAs, glucagon‐like peptide 1 receptor agonists; FT, free testosterone; SHBG, sex hormone‐binding globulin; TT, total testosterone.

Concerning the effects of GLP‐1RAs on serum hormone levels, subjects treated with GLP‐1RAs showed greater increase in TT levels than controls (sMD 0.79; 95% CI 0.12−1.47; *p* = 0.02; *I* ^2^ = 84%). Subgroup analysis demonstrated a significant difference when GLP1‐RAs were compared with other antidiabetics but not with hormone replacement treatment (*p* = 0.001) (Figure 9A). On the other hand, the administration of GLP‐1RAs drugs led to changes in FT and SHBG levels comparable with the control group (Figure 9B,C).

**FIGURE 10:**
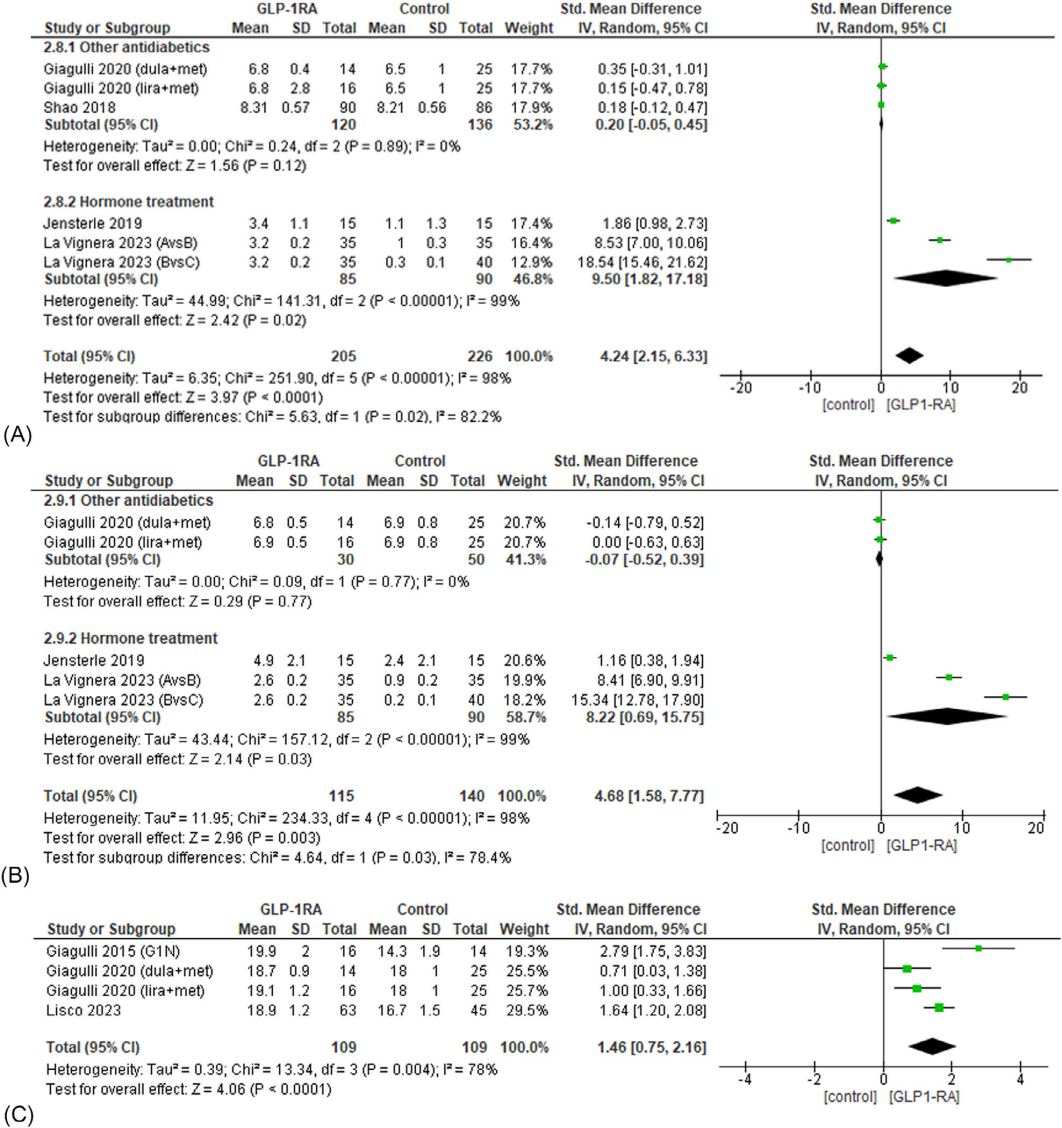
Subgroup analysis and forest plot of LH levels (A), FSH levels (B), and IIEF scores (C) in patients treated with GLP‐1RAs compared with controls. FSH, follicle‐stimulating; GLP‐1RAs, glucagon‐like peptide 1 receptor agonists; IIEF, International Index of Erectile Function; LH, luteinizing hormone.

Interestingly, the treatment with GLP‐1RAs drugs showed more pronounced effects than controls on gonadotropin levels and erectile function indexes (Figure 10A–C). Indeed, in four studies^22^, ^26^, ^27^, ^28^ patients treated with GLP‐1RAs showed significantly higher levels of serum LH compared with the control group (sMD 4.24; 95% CI: 2.15−6.33; *p* < 0.0001; *I* ^2 ^= 98%). Notably, subgroup analysis demonstrated that the effect was significant only when the GLP‐1RAs treatment was compared with hormone replacement treatment (*p* = 0.02).

Similarly, three studies^26^, ^27^, ^28^ showed a greater increase in FSH levels in patients undergoing GLP‐1RAs treatment (sMD 4.68; 95% CI: 1.58−7.77; *p* = 0.003, *I* ^2^ = 98%), that remained significant only when control group was represented by subjects under hormone therapy (*p* = 0.03).

Finally, the effects on erectile function was investigated in three studies.^23^, ^24^, ^26^ The administration of GLP‐1RAs drugs led to a greater increase in IIEF score compared to controls (sMD 1.46; 95% CI 0.75−2.16; *p* = 0.004; *I* ^2 ^= 78%), which remained significant after the exclusion of the study by Giagulli et al.^26^ (sMD 1.16; 95% CI: 0.57−1.75; *p* = 0.0001; *I* ^2 ^= 66%). None of the included studies had used hormone therapy as a control, so subgroup analysis was not performed.

## 4 DISCUSSION

Our systematic review and meta‐analysis showed that the administration of GLP‐1RAs in men suffering from overweight/obesity may significantly improve testicular function by decreasing body weight. This is supported not only by the favorable effect of these drugs on TT, FT, and SHBG levels, but also by the significant increase in gonadotropin levels, proving that the suppression on the hypothalamic−pituitary−gonadal (HPG) axis exerted by excess weight is reduced. Indeed, as stated before, systemic inflammation and aromatase activity impair gonadotropic secretion by negative feedback.^3^, ^9^, ^30^ Interestingly, meta‐regression showed a significant correlation between the increase in SHBG levels and the decrease in HbA1c levels, but no association was found with weight reduction. This finding is unexpected, since overfeeding^31^ and obesity^32^ are frequently associated with low SHBG levels. Nevertheless, it might support a possible role in the regulation of insulin resistance by SHBG independently from weight loss, as recently suggested by several studies in men^33^, ^34^, ^35^ and women.^36^ Appropriately structured studies are obviously required to evaluate this hypothesis.

The strong association between weight loss and increased TT and FT levels found in the meta‐regression analysis supports its positive impact on the pathophysiology of functional hypogonadism induced by excess weight. However, no definitive conclusion can be drawn on a potential direct effect of GLP‐1 on the testicular GLP‐1R. To delve deeper into this matter, we analyzed the effect of GLP‐1RAs on serum androgen levels compared to other drugs, but—despite e more favorable effect on BMI—no significant difference emerged. Notably, the majority of the included studies had an observational design and high heterogeneity in the choice of alternative treatments in the control groups, making the interpretation of results complex. Specifically, five studies included only subjects with DM2 treated with metformin, and no weight‐lowering intervention was used as comparison.^22^, ^23^, ^24^, ^25^, ^26^ In this regard, the weight‐lowering effect of GLP‐1RAs seems to be generally more pronounced in subjects without diabetes (6.1%−17.4%) compared to men with DM2 (4%−6.2%).^37^ Indeed, the great efficacy of GLP‐1RAs in obese men without diabetes was confirmed recent meta‐analysis conducted on 14 RCTs with more than 10,000 subjects without diabetes (weight loss in GLP‐1RAs consumers compared with placebo consumers: MD, −8.77 kg; 95% CI: −10.98, −6.56; *P* < 0.01; *I* ^2^ = 100%).^38^ Conversely, the favorable effect on weight appears much less evident in subjects with diabetes, in whom it often appears insignificant.^39^ Several hypotheses have been made, including the weight gain effect of concomitant antidiabetic drugs (e.g., insulin and sulfanilureas), reduced dietary adherence due to fear of hypoglycemia, reduced glycosuria achieved by improved glycemic compensation, and alterations in the microbiome in subjects with DM2.^37^ It is possible that such differences also translate to testicular function in men treated with GLP‐1RAs with or without diabetes and should be investigated.

On the other hand, La Vignera et al. compared the effects of GLP‐1RA (Group B) with gonadotropin (Group A) or testosterone replacement therapy (Group C) in nondiabetic obese men suffering from hypogonadism,^28^ whereas Jensterle et al. compared the effect of GLP‐1RAs and transdermal testosterone in a group of hypogonadic obese subjects independently from their glycemic status.^27^ Additionally, in the study by Shao et al., the control group underwent treatment with glimepiride.^22^ Glimepiride is a drug belonging to the class of sulphanylureas, which has the well‐known side effect of weight gaining.^6^ This could lead to a subsequent further suppression on the HPG axis, thus overestimating the favorable effect on testicular function in the treatment group. Moreover, the lack of randomization leads to a significant selection bias associated with the presence of comorbidities that influenced treatment choice. Indeed, GLP‐1RAs are the first‐choice of treatment in patients with obesity and high cardiovascular risk,^40^, ^41^ necessarily leading to a significant imbalance in patient characteristics at baseline in observational studies. It is obvious that randomized, controlled trials in which the action of GLP‐1RAs is compared with an intervention aimed at achieving superimposable weight loss are needed to determine whether the improvement in the gonadal function is exclusively attributable to a direct action of these drugs.

Interestingly, when compared with other treatment options, GLP‐1RAs showed a more pronounced increase in gonadotropin levels. Despite the possibility of this event being uniquely dependent on the change in BMI, preclinical studies show that GLP‐1 also increases the expression of kisspeptin in hypothalamic neuron,^42^ and secretion of LH by the pituitary,^43^ thus suggesting a direct stimulation of the HPG axis. Further studies on humans are undoubtedly needed to confirm this hypothesis.

Similarly, men undergoing GLP‐1RAs therapy showed a greater increase in IIEF score compared to patients taking other antidiabetic drugs. Once again, a significant decrease in BMI could largely explain this observation. Nevertheless, GLP‐1RAs role in men suffering from ED still need to be studied in more depth. Indeed, vasculogenic ED is a known cardiovascular risk factor in patients with diabetes,^44^ and may be a useful marker of advanced atherosclerosis.^45^ Men with ED may benefit from therapy with GLP‐1RAs because it could prevent future major cardiovascular events; it also has a specific role in improving erectile function, but confirmatory studies are required to turn this advice in a recommendation.

Regarding semen quality, only two studies with very different design have analyzed the effects of GLP‐1RAs on sperm parameters^28^, ^29^ and could not be included in the quantitative analysis. In the randomized controlled double‐blind trial by Andersen et al.,^29^ a group of 56 men followed an hypocaloric diet (800 kcal/die) for 8 weeks, which led to important weight loss (−16.5 kg) and improvement in several semen parameters (1.49‐fold increase in sperm concentration and 1.41‐fold increase in sperm count). Following randomization, patients were assigned to four different groups of maintenance therapy: placebo and habitual activity, placebo and exercise training, liraglutide and habitual activity, and liraglutide and exercise training. After 52 weeks, only the patients managed to maintain the weight they lost also maintained the improvement in semen quality, regardless of the assigned treatment group. In the second study (that was already included in the present meta‐analysis), La Vignera et al.^28^ enrolled 110 obese males affected by functional hypogonadism; they were assigned to three different treatment group, each one in association with an hypocaloric diet of 1400–1800 kcal/die: gonadotropins (urofollitropin 150 UI 3 times a week + human chorionic gonadotropin 2000 UI 2 times a week), GLP‐1RAs (liraglutide 3 mg/die), and testosterone (testosterone gel 2% 60 mg/die). Subsequently, after 4 months of treatment, semen analysis was performed in patients in the gonadotropins and GLP‐1RAs groups. The liraglutide therapy led to a significant increase in all conventional sperm parameters; the observed results are better than what was observed in patients treated with gonadotropins. Additionally, it should be noted that patients treated with GLP‐1RAs showed a sharp decrease in body weight (−10.3%) and BMI (−16.7%) that was not achieved in the other treatment groups. Since a multivariate analysis was not performed, whether this is a direct effect of GLP‐1RAs at the testicular level or direct effect mediated by weight loss cannot be determined. Therefore, these data should be taken with caution, and overall, no definitive conclusions can be drawn.

In conclusion, the results of our systematic review and meta‐analysis suggest a possible role for GLP‐1RAs in the therapy of functional hypogonadism related to overweight promoting rapid weight loss in combination with other lifestyle interventions. However, the overall effect found on testosterone levels is statistically significant, but modest, especially if the source studies of high heterogeneity are removed. The clinical significance of this increase, therefore, remains to be clarified. Similarly, the observed increase in gonadotropins after treatment is slight and influenced mainly by a single study but seems to suggest a potential positive effect in correcting the suppression of the HPG axis involved in obesity‐related functional hypogonadism. The great heterogeneity of the included studies, the lack of randomization and adequate control groups, and the low number of patients involved does not allow for the demonstration of a direct testicular activity of GLP‐1RAs. Well‐designed clinical trials are therefore needed to further investigate the effects of GLP‐1RAs on the male reproductive system.

## AUTHOR CONTRIBUTIONS

**Gianmaria Salvio and Alessandro Ciarloni**: Writing; reviewing; editing; investigation; conceptualization; formal analysis. **Nicola Ambo, Monia Bordoni, Michele Perrone, and Silvia Rossi**: Investigation; review and editing. **Giancarlo Balercia**: Review; editing and supervision.

## CONFLICT OF INTEREST STATEMENT

The authors declare no conflict of interest.

## Supporting information



Supporting information

## Data Availability

The data that support the findings of this study are available from the corresponding author upon reasonable request.
