# Supplementary material for: Effects of glucagon‐like peptide 1 receptor agonists on testicular dysfunction: A systematic review and meta‐analysis
Source: Andrology. 2025 Mar 19;13(8):2022–34. doi: 10.1111/andr.70022 (PMC12569732; doi:10.1111/andr.70022)
Supplement: Supplementary file 1 — Supporting information [file ANDR-13-2022-s001.docx]

Supporting materials

Complete search strategy

The following query strings were used: ("glucagon like peptide 1"[MeSH Terms] OR "glucagon like peptide 1"[All Fields] OR "glp 1"[All Fields] OR ("liraglutid"[All Fields] OR "liraglutide"[MeSH Terms] OR "liraglutide"[All Fields] OR "liraglutide s"[All Fields]) OR ("exenatide"[MeSH Terms] OR "exenatide"[All Fields] OR "exenatide s"[All Fields]) OR ("dulaglutide"[Supplementary Concept] OR "dulaglutide"[All Fields]) OR ("semaglutide"[Supplementary Concept] OR "semaglutide"[All Fields]) OR ("lixisenatide"[Supplementary Concept] OR "lixisenatide"[All Fields]) OR ("glucagon like peptide 1"[MeSH Terms] OR "glucagon like peptide 1"[All Fields] OR "glucagon like peptide 1"[All Fields]) OR "GLP"[All Fields]) AND ("hypogonad"[All Fields] OR "hypogonadal"[All Fields] OR "hypogonadic"[All Fields] OR "hypogonadism"[MeSH Terms] OR "hypogonadism"[All Fields] OR "hypogonadisms"[All Fields] OR ("testosterone"[MeSH Terms] OR "testosterone"[All Fields] OR "testosteron"[All Fields] OR "testosterones"[All Fields] OR "testosterone s"[All Fields]) OR "testic*"[All Fields] OR ("sperm s"[All Fields] OR "spermatozoa"[MeSH Terms] OR "spermatozoa"[All Fields] OR "sperm"[All Fields] OR "sperms"[All Fields]) OR ("infertiles"[All Fields] OR "infertilities"[All Fields] OR "infertility"[MeSH Terms] OR "infertility"[All Fields] OR "infertile"[All Fields] OR "infertility s"[All Fields])) (Pubmed and adapted for Embase), “( TITLE-ABS-KEY ( glp-1 ) OR TITLE-ABS-KEY ( liraglutide ) OR TITLE-ABS-KEY ( exenatide ) OR TITLE-ABS-KEY ( dulaglutide ) OR TITLE-ABS-KEY ( semaglutide ) OR TITLE-ABS-KEY ( lixisenatide ) OR TITLE-ABS-KEY ( glucagon-like AND peptide 1 ) OR TITLE-ABS-KEY ( glp ) ) AND ( TITLE-ABS-KEY ( hypogonadism ) OR TITLE-ABS-KEY ( testosterone ) OR TITLE-ABS-KEY ( testic* ) OR TITLE-ABS-KEY ( sperm ) OR TITLE-ABS-KEY ( infertility ) OR TITLE-ABS-KEY ( erectile AND dysfunction ) ) AND ( LIMIT-TO ( DOCTYPE , "ar" ) )” (Scopus).

Table S1. Full texts assessed for eligibility and excluded, with reasons

| **Source** | **First author, year** | **Title** | **Journal** | **Reason** |
| --- | --- | --- | --- | --- |
| Scopus | Sun S, 2021 | Acute glucagon-like peptide-1 infusion has no effects on sex hormones in healthy men | Chinese Journal of Endocrinology and Metabolism | Editorial, no data available |
| Embase | Yin D, 2023 | Effects of glucagon-like peptide-1 on male reproductive function | Chinese Journal of Endocrinology and Metabolism | Review, no data available |
| Pubmed | Wägner AM, 2018 | Effect of liraglutide on physical performance in type 2 diabetes (LIPER2): A randomised, double-blind, controlled trial | Diabetes & Metabolism | Other topic, primary outcome not reported |
| Scopus | Jeibmann A, 2005 | Glucagon-like peptide-1 reduces the pulsatile component of testosterone secretion in healthy males | European Journal of Clinical Investigation | Other topic, primary outcome not reported |
| Pubmed | Andersen E, 2022 | Sperm count is increased by diet-induced weight loss and maintained by exercise or GLP-1 analogue treatment: a randomized controlled trial | Human Reproduction | No data available |
| Scopus | Able C, 2024 | Prescribing semaglutide for weight loss in non-diabetic, obese patients is associated with an increased risk of erectile dysfunction: a TriNetX database study | International Journal Of Impotence Research | No data available |
| Scopus | Izzi-Engbeaya C, 2020 | Effects of glucagon-like peptide-1 on the reproductive axis in healthy men | Journal of Clinical Endocrinology and Metabolism | Acute GLP-1 administration, primary outcome not reported |
| Scopus | Defeudis G, 2022 | The Role of Antihyperglycemic Drugs and Diet on Erectile Function: Results from a Perspective Study on a Population with Prediabetes and Diabetes | Journal of Clinical Medicine | Primary outcome not reported |
| Embase | Bobkov DN, 2021 | Ejaculate quality indicators of men with asthenozoospermia and asthenoteratozoospermia by treatment of obesity with liraglutide. | Obesity and Metabolism | No data available |
| Scopus | Bajaj HS, 2021 | Erectile function in men with type 2 diabetes treated with dulaglutide: an exploratory analysis of the REWIND placebo-controlled randomised trial | Lancet Diabetes and Metabolism | Primary outcome not reported |
| Pubmed | Madsbad S, 2021 | Dulaglutide for erectile dysfunction in type 2 diabetes | Lancet Diabetes and Metabolism | Editorial, no data available |


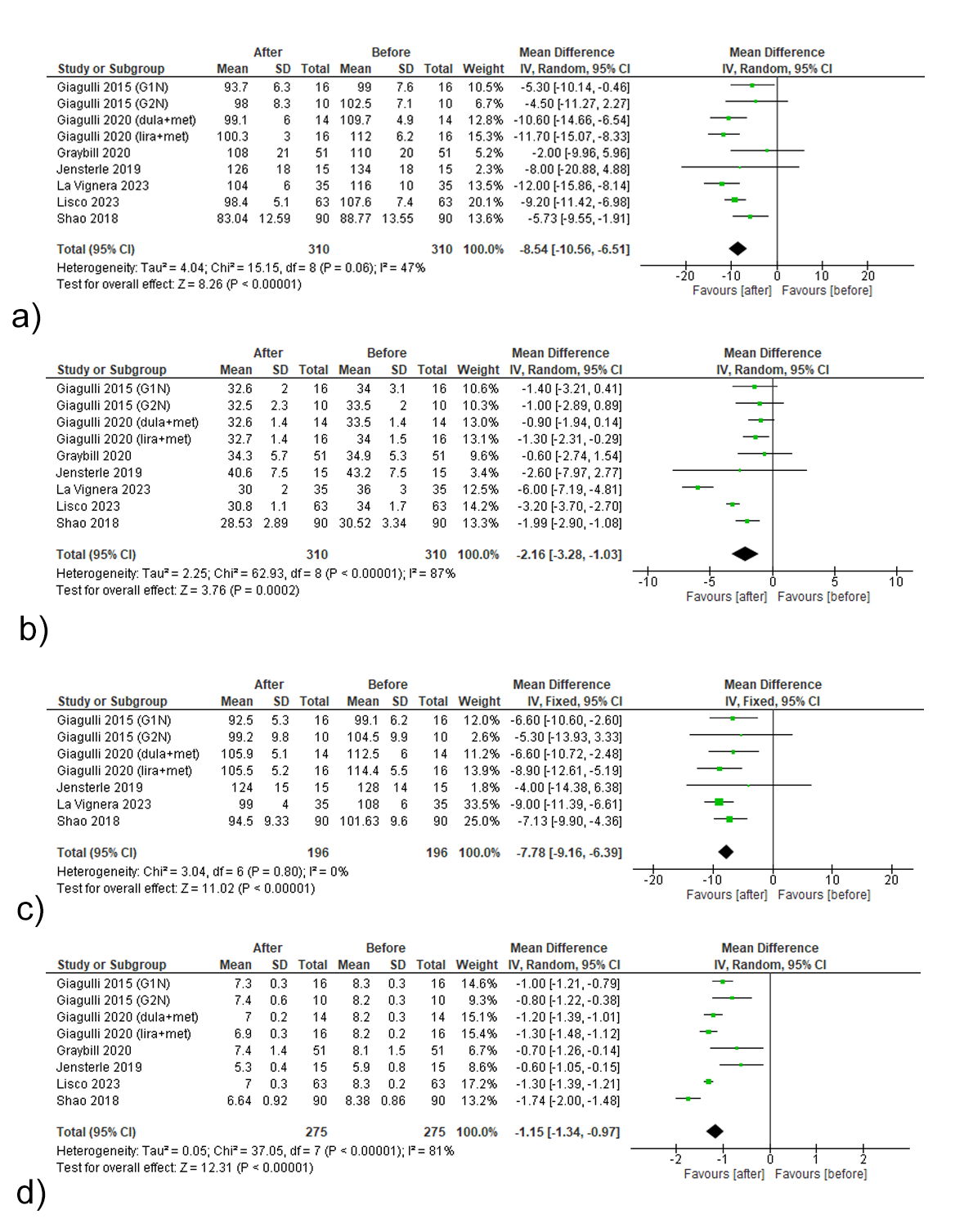


Figure S1: Forest plot of weight (a), BMI (b), WC (c), and HbA1c (d) levels in patients before and after GLP-1RAs administration. BMI = body mass index; GLP-1RAs = glucagon-like peptide 1 receptor agonists; HbA1c = glycated hemoglobin; WC = waist circumference.


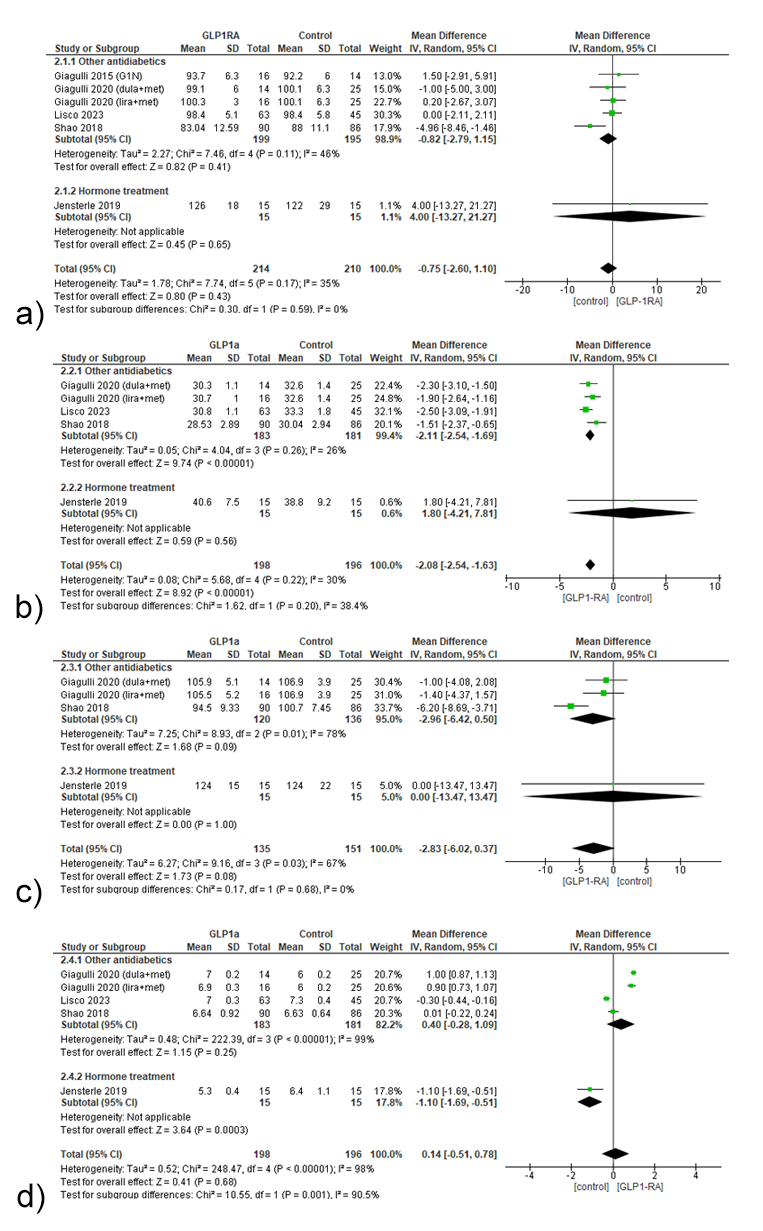


Figure S2: Forest plot of weight (a), BMI (b), WC (c), and HbA1c (d) levels in patients treated with glucagon-like peptide 1 receptor agonists (GLP-1RAs) compared with controls. BMI = body mass index; GLP-1RAs = glucagon-like peptide 1 receptor agonists; HbA1c = glycated hemoglobin; WC = waist circumference.
